# Primary care physician beliefs and practices regarding blood pressure measurement: results from BP-CHECK qualitative interviews

**DOI:** 10.1186/s12875-022-01950-1

**Published:** 2023-01-25

**Authors:** Clarissa Hsu, Laurel Hansell, Kelly Ehrlich, Sean Munson, Melissa Anderson, Karen L. Margolis, Jennifer B. McClure, Yoshio N. Hall, Matthew Thompson, Dwayne Joseph, Beverly B. Green

**Affiliations:** 1 Kaiser Permanente Washington Health Research Institute, 1730 Minor Av. Suite 1600, Seattle, WA 98101 USA; 2 Department of Human Centered Design and Engineering, University of Washington, Seattle, WA USA; 3 HealthPartners Institute, Minneapolis, MN USA; 4 Kaiser Permanente Bernard J Tyson School of Medicine, Pasadena, CA USA; 5 Kidney Research Institute, Department of Medicine, University of Washington, Seattle, WA USA; 6 Department of Family Medicine, University of Washington, Seattle, WA USA; 7 Washington Permanente Medical Group, Seattle, WA USA

**Keywords:** Blood pressure, Diagnosis, Measurement, Provider attitudes, Hypertension, Kiosk, Home, Ambulatory, Primary care, Out-of-clinic

## Abstract

**Background:**

Early identification and control of hypertension is critical to reducing cardiovascular disease events and death. U.S. Preventive Services Task Force guidelines recommend health care professionals screen all adults for hypertension, yet 1 in 4 adults with hypertension are unaware of their condition. This gap between guidelines and clinical practices highlights an important opportunity to improve blood pressure (BP) screening and hypertension diagnosis, including measurement outside of clinic settings. To identify targets for future diagnostic interventions, we sought to understand primary care physicians' (PCPs) beliefs and practices regarding use of common forms of BP measurement.

**Methods:**

Study participants were PCPs (*N* = 27) who had patients enrolled in the BP-CHECK trial. The trial compared the accuracy of 24-h ambulatory BP monitoring (ABPM) to: 1) clinic-based monitoring, 2) home BP monitoring (HBPM), or 3) use of a BP kiosk in clinics or pharmacies. Physicians were interviewed by phone and compensated for their participation. Interviews were recorded, transcribed, and analyzed using a template analysis approach.

**Results:**

Overall, PCPs preferred and trusted clinic BP measurement for diagnosing hypertension, particularly when measured with a manual sphygmomanometer. Concerns with HBPM included the belief that patients did not follow protocols for rest and body positioning at home, that home machines were not accurate, that home BPs could not be entered into the medical record, and that HBPM would make some patients anxious. Issues regarding kiosk measurement included beliefs that the public setting created stress for patients, that patients did not follow resting protocols when using kiosks, and concerns about the maintenance of these machines. ABPM was recognized as highly accurate but was not perceived as accessible. Additionally, some PCPs found it challenging to interpret the multiple readings generated by ABPM and HBPM, especially when these readings differed from clinic BPs.

**Conclusions:**

Our findings suggest that both additional physician education and training and investments in equipment and system-level processes are needed to increase the acceptance and utilization of out of office BP measurement for identification and treatment of hypertension. These changes are needed to improve ensure everyone in the U.S receive optimal care for hypertension.

**Trial registration:**

ClinicalTrials.gov NCT03130257. Initial registration date: 4/21/2017.

**Supplementary Information:**

The online version contains supplementary material available at 10.1186/s12875-022-01950-1.

## Introduction

Hypertension is the most common cause of avoidable death and disability globally [1]. Early identification and control is critical to reducing cardiovascular disease events and death [2]. Hypertension also is the most common diagnosis at clinic visits and accounts for more than half of adult diagnoses for a chronic condition [3]. Despite this prevalence, more than 1 in 4 adults with hypertension are unaware they have this life-threatening condition [4, 5].

Currently, the U.S. Preventive Services Task Force (USPSTF) guidelines recommend screening all adults aged 18 years or older for hypertension (an “A” recommendation, strong evidence of benefit) [6, 7]. The recommended initial screen is clinic-based measurement using a manual or automated sphygmomanometer. For patients with high screening blood pressure (BP), the USPSTF guidelines recommend follow-up diagnostic testing outside the clinic, preferably 24-h ambulatory BP monitoring (ABPM) or home BP monitoring (HBPM) with readings averaged over an extended time (24 h to several days). Similarly, the American College of Cardiologists and American Heart Association hypertension guidelines recommend out-of-clinic BP monitoring by ABPM or HBPM before diagnosing hypertension [8].

These guidelines contrast with current practice for many primary care physicians (PCPs) who rely almost exclusively on clinic-based BP measurement using manual or automated sphygmomanometers and no averaging of readings [9]. However, several nonclinic options now exist for measuring BP, including home monitors, automated BP kiosks in clinics and pharmacies, and ABPM. Each of these methods can be used to supplement or replace clinic BP monitoring. However, their utility may not be fully recognized or embraced by physicians in the U.S. for diagnosing hypertension. Thus, understanding physicians’ beliefs and practices about different BP measurement methods is important to understanding and addressing this potential gap in care in the U.S.

The literature on health care professionals’ knowledge and attitudes regarding BP measurement, especially when used for diagnosis or taken outside of a clinic, is limited. Even fewer studies focus on knowledge and beliefs of health care professionals in the U.S., which has systemic constraints on out-of-clinic BP measurement [10] such as low reimbursement rates for ABPM [11]. This paper seeks to address these important issues from the perspective of PCPs in the U.S. as part of Blood Pressure Checks for Diagnosing Hypertension (BP-CHECK), a randomized controlled diagnostic trial that compared the performance and acceptability of home, clinic, and kiosk-based BP measurement against mean daytime BP measured by ABPM [12, 13]. A key finding of the study was that HBPM diagnostic testing was significantly more likely to correctly identify individuals with hypertension than follow-up clinic BP measures, reinforcing the importance of using ABPM or HBPM for diagnosing hypertension. As part of the BP-CHECK study, we surveyed health professionals (medical assistants [MAs], nurses, physicians, physician assistants, and nurse practitioners) at the beginning of the study to assess their knowledge, beliefs, and practices for diagnosing hypertension. Quantitative findings demonstrated that health professionals had significant knowledge gaps, with beliefs and practices that were not well aligned with evidence-based recommendations.

This qualitative study interviewed physicians whose patients participated in the BP-CHECK study about their beliefs and practices related to using different BP measurement methods and their experience with using HBPM and ABPM for diagnosing hypertension. Our aim was to shed light on barriers and potential facilitators for improving hypertension diagnosis.

## Methods

### Goal

The goal was to assess primary care physicians’ (PCPs) attitudes and practices after being exposed to a variety of BP measurement options for hypertension diagnosis. (See Supplemental Materials for Interview Guide). The Kaiser Permanente Washington Health Research Institute Institutional Review Board approved this study and all participants provided verbal consent.

### Sampling and recruitment

**Fig. 1:**
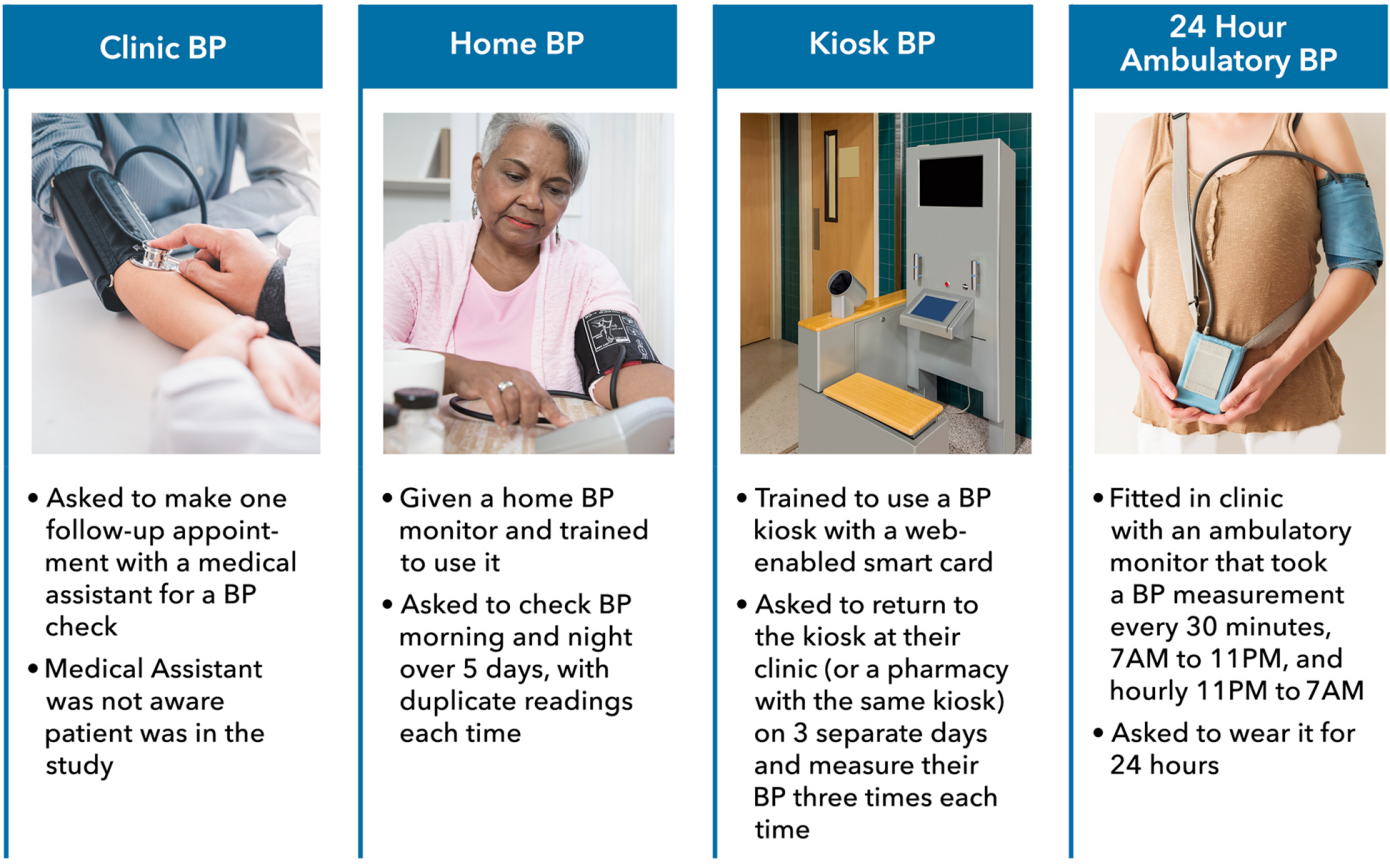
Summary of BP-CHECK measurement methods

This qualitative study included PCPs (*N* = 27) from 12 clinics who had one or more patients enrolled in the BP-CHECK study. Patient participants in the BP-CHECK study were all members of the Kaiser Permanente health care system in Washington state. Patients were randomized to use either home, clinic, or kiosk-based BP monitoring. After completing their assigned measurement protocol, each participant completed ABPM (Fig. 1). ABPM results were shared with the participants and entered into their electronic health record (EHR) and routed to the patient’s PCP with a recommendation to follow up with the patient if ABPM results were positive for hypertension (defined as daytime ABPM mean systolic ≥ 135 mmHg or mean diastolic ≥ 85 mmHg). PCPs were sent reminders until they viewed their patients’ results. As part of the BP-CHECK study, PCPs were invited to attend brief informational meetings at each clinic before the trial started, facilitated by the study leader, a physician researcher. Providers were told that all study participants would be asked to complete an ABPM. The researcher shared best practices for interpreting ABPM. PCP interviews took place shortly after the study finished recruiting patients from the clinic where the PCP practiced.

The interviews analyzed for this paper focused on PCPs at Kaiser Permanente Washington who received ABPM results for a BP-CHECK study participant, to ensure they had some experience with this testing methodology. A purposive sampling matrix was used to prioritize recruitment of PCPs who received 2 or more ABPM patient reports with the goal of approximately 75% of participating PCPs receiving 2 or more ABPM reports. Invitations to participate and study information summaries were sent to PCPs via email, followed by at least 3 attempts at phone outreach if there was no response.

### Data collection

Phone interviews took place between March 2018 and April 2019 and were conducted by experienced qualitative researchers (SE and LH) who followed a semi-structured interview guide. Participants provided verbal consent and were offered $50 as thanks for participation. Interviews were recorded and transcribed for analysis.

### Data analysis

Transcripts were coded by three qualitative researchers (SE, LH & CH). Using a template analysis approach [14], the team identified a priori and emergent themes/codes from a diverse subset of transcripts to serve as the basis for a draft code list and code book. Each coder then independently coded the same interviews and noted suggested revisions and additions to the code book. The coding team then met to discuss codebook revisions, address questions, and compare and reconcile coding. This discussion consensus process was repeated until the code book had stabilized (i.e., few revisions were suggested) and a high rate of agreement was reached on the application of the codes and code definitions. Once the codebook was finalized, the researchers coded the remainder of the transcripts independently and met regularly to discuss emergent questions and confirm coding accuracy and consistency.

Once coding was complete, data were pulled by code and selected codes were analyzed further. This analysis was summarized in one or more analytic coding memos [15]. These coding memos served as the basis for the findings presented.

## Results

The 27 study participants were demographically diverse (Table 1). All but two specialized in family medicine. On average, they had been in medical practice for 16 years (range = 4–38 years). Most practiced medicine full-time.

**Table 1:** Participant characteristics

	*N* = 27
**Age in years**	
25–34	3
35–44	9
45–54	5
55–64	4
65–74	2
Missing ^a^	4
**Sex**	
Male	6
Female	19
Missing ^a^	2
**Race/Ethnicity**	
BIPOC	7
White	20
Hispanic	0
**Avg years practicing** (range)	16 (4–38)
**Avg years at KPWA** (range)	9 (1–35)
**Avg FTE**	0.9
**Specialty**	
Family medicine	25
General internal medicine	2

*FTE* Full time equivalent, *KPWA* Kaiser Permanente Washington, *BIPOC* Black, Indigenous, People of Color, which includes race and ethnicity categories other than White such as American Indian or Alaska Native, Asian, Black or African American, or Native Hawaiian or other Pacific Islander

^a^ This item was missing for several individuals who were not asked the demographic questions during the interview and were not responsive to follow-up attempts to get these data

Key themes emerged related to physician beliefs and practices regarding BP screening and measurement for diagnosis. These themes were organized around the measurement methods used in the trial. Overarching themes are discussed at the end of the results section.

### Clinic blood pressure measurement

Most PCPs trusted and relied primarily on clinic measurement to make a hypertension diagnosis and tended to rely on BPs taken by an MA or nurse using manual techniques (auditory measurements taken with a stethoscope, hand-inflated cuff, and aneroid BP monitor) over automated BP monitor measurements. PCPs explained that reliance on manual BP readings is a product of their training.

*So, in clinic we always use the manual because I guess from my training that was considered the gold standard.* (Clinic 6, PCP2)

*I would say a manual monitor [is more accurate]—at least this is what I'm taught, and this is kind of what I use in my practice—the manual monitor's the most effective. (Clinic 4, PCP20)*

Some stated that they prefer clinic readings—either manual or automated—over home and kiosk measurements for diagnosis because they felt more confident that the patient had rested the recommended 5 min before having their BP taken and that all the procedures were correct, including body positioning and support.

*I have a preference to bring them into my clinic to have a manual blood pressure check by my MA, because I know they have them rest for five minutes before they check the blood pressure, make sure their arms aren't crossed and make sure the arm's rested and held at heart level when they're checking it, because those are sort of the norms we indicate to our MAs when they have a protocol to recheck it, if it's elevated beyond…140/90. If it's elevated beyond that, they usually recheck it in about five minutes and then notify us.* (Clinic 4, PCP16)

Although many PCPs saw clinic BP measurement as accurate, many also recognized that it was not the most patient-centered option, especially because of the inconvenience of coming into the clinic.

*Well, I think the only thing I hear patients complaining about is having to come in, if they weren't otherwise, needing to make the time, right? So, it's obviously extra work for people to come in through traffic, park and come in and then sit and wait for their blood pressure to be checked, so that would be one obvious barrier.* (Clinic 1, PCP7)

*I think having patients come in for a frequent follow up, whether it's a doctor's visit or a blood pressure check visit, is not very patient centered because it's hard for people to come in. In my clinic, I work in a particular…clinic that's extremely non-patient-friendly for access. We have very poor parking…So it's just hard for people to come in.* (Clinic 2, PCP19)

*Some patients live far away from clinics so coming to see us even without a copay is really a barrier because it's a 90-minute drive for them.* (Clinic 4, PCP10)

Others acknowledged that automated machines might be more accurate but stated that they did not have access to these machines. While all clinics had at least one automated BP measurement device, for some clinics the number of devices was not sufficient to make them accessible for consistent use.

*I know there have been studies about the automatic cuff being more accurate than the humans. …I'm not sure, I haven’t had access to an automatic cuff since forever… Everything's manually, no automatic cuffs. So, I'd like to do that, I think that would be super helpful, but it's not an option.* (Clinic 4, PCP12)

### Home blood pressure measurement

PCPs viewed home monitors as useful tools but did not trust them for diagnosis. They voiced concerns about the accuracy of home monitoring, including the belief that current diagnosis thresholds were based on clinic BPs and therefore may not be accurate for HBPM and that patients may not be resting or seated in the optimal position when taking their BP. The most common concern about HBPM was that home machines were not accurate and needed to be tested against a clinic measurement.

*I'm less comfortable with the kiosk and the home blood pressure reading…if someone's truly been resting or their position and things like that. And then oftentimes if someone brings in home readings, I don't know if their machine is calibrated or something like that, so it makes me a little more uncomfortable.* (Clinic 1, PCP5)

Another challenge of HBPM is that, at the time of the interviews (2018- 2019), the health care system did not consider HBPM an acceptable measure because there was no way to enter readings into the EHR and it did not count for quality measures. These circumstances have since changed due in part to COVID-19 and HBPM measures can at least temporarily be used for quality measures.

*I would be much more open to using the home numbers, but for whatever reason they have not been considered a legitimate use of the numbers. So, we'll keep getting reports saying people are out of control and [the EHR will do] automatic outreach and things like that... so it's really just from a practical standpoint, [we] can't use the home numbers because they do not translate into the [health care system’s] goal setting. That's why I use the clinic numbers …. There's a certain kind of tail wagging the dog … I recognize, but somebody emails their home numbers, and they are good, they're still going to show up on a report until they come in and get a blood pressure that looks good.* (Clinic 1, PCP7)

Although PCPs said they were uncomfortable using HBPM to diagnose hypertension, they often acknowledged that home BP monitors were more patient centered because of the convenience they offer.

*But I do think being able to check your blood pressure at home is probably for most people the most convenient thing to do.* (Clinic 3, PCP12)

*It's just easy. You don't have to leave the home to go anywhere, which I think is what people prefer, I would prefer that. Especially if you're older, which most of my patients with hypertension are, or a lot of them are. Transportation can be an issue.* (Clinic 1, PCP5)

*That's the other thing about the home monitoring. I just feel that works so well for a lot of my patients to just check it at home and have them come in once and make sure the blood pressure cuff is accurate and then we can just titrate meds, do all this virtually. To me that just seems like much better.* (Clinic 2, PCP9)

PCPs also worried that HBPM could make some patients anxious if they were getting high readings. They worried this would lead to a vicious cycle in which a patient would have increased anxiety about high BP readings, which would in turn cause elevated BP readings.

*There is a subset who take a reading and they get anxious and then a minute later they take another reading and it's higher, another minute later they take another reading and it's higher, and they just work themselves up into a complete panic. So [there is a] slice of patients with hypertension and anxiety for whom I'd specifically say I do not recommend that you monitor your blood pressure at home, but that's the minority.”* (Clinic 2, PCP13)

*But sometimes the patient is too much like anxious, they are pretty much checking the blood pressure 16 or 17 times per day, and I don't think that is good. …You don't want to get them in a panic….* (Clinic 6, PCP3)

Only one PCP mentioned multiple data points as an advantage of HBPM even though guidelines recommend collecting BPs over several days and averaging them.

### Kiosk blood pressure measurement

PCPs had several concerns about BP kiosks in pharmacies or clinic waiting rooms. First, PCPs were concerned about reliability, maintenance, the setting in which BP kiosks are located, and cuff size limitations.

*The other one I really don't like is the kiosk ones, just because I find that people say, "I checked the blood pressure at the kiosk and it was high," but they were using it at the store, and they were walking around, and they weren't doing it properly. Or they're obese, and their arm size probably isn't a one-size-fit-all cuff. I think that is my least favorite.* (Clinic 6, PCP3)

They also felt it would be hard for patients to follow standard procedures to rest before taking their BP when using a kiosk.

*I do refer patients that don't have a cuff to go use those [kiosks]. I think they're another good tool to have. My only concern with them is that often it's hard for people to go sit down for five minutes before they do a blood pressure reading. If you're like at work, you're more in a get-in, get-out attitude. I have no real data to support that, but I tend to believe the readings that we were getting. I just always like to check if they've actually been seated for five minutes before they get their blood pressure checked.”* (Clinic 2, PCP17)

Several PCPs reported that kiosks tended to give higher BPs readings, which had both benefits and challenges.

*So, to me, store kiosk blood pressure cuffs are the same as home cuffs in the sense that I don't know how accurate they really are. I'm sure it's variable. But at the same time, I think it helps because if they're measuring high and I'm measuring an office high, I think it helps reinforce the people who aren't convinced that they really have high blood pressure, who think it's only high in the office. So, I do tell people to check outside the office and let me know what reading they're getting, but I'm not 100% confident in them.* (Clinic 6, PCP1)

### 24-h Ambulatory blood pressure measurement

PCPs reported having a high level of confidence in the overall accuracy and clinical value of ABPM results.

*I would say it [the 24-hour monitor] is just a more robust set of data, so when you're doing ambulatory blood pressure monitoring, that's the gold standard.* (Clinic 1, PCP7)

*I guess with the 24-hour monitoring, I do feel more confident telling a patient this really helps confirm your diagnosis being hypertensive and indicates that treatment would be helpful.* (Clinic 3, PCP12)

A few had ordered an ABPM to confirm a hypertension diagnosis prior to the BP-CHECK study.

*Typically, in patients that do not currently carry a diagnosis, but I have an elevated reading, and are uncertain or unconvinced of whether they need medication. So, I'll use it as a tool to confirm diagnosis and establish a need for medication* (Clinic 2, PCP13)

Many PCPs reported that ABPM was a good way to resolve situations where patients presented with unclear BPs readings in clinic, inconsistencies between clinic readings and home readings or when patients were resistant to diagnosis and/or treatment.

*I guess with the 24-hour monitoring, I do feel more confident telling a patient this really helps confirm your diagnosis being hypertensive and indicates that treatment would be helpful.* (Clinic 3, PCP12)

*So, I guess if they're just in the clinic and they're someone that's had consistent hypertension, I tend to rely more on the manual readings. If it's someone that's had really labile blood pressure, that's when I would usually use the 24-hour blood pressure monitoring.* (Clinic 2, PCP 17)

*Well, I would like to be able to order a 24-hour home monitoring test whenever there's a lack of clarity behind diagnosis. It happens a lot where I notice that the blood pressure trend in the office has been high, and I suspect that it more likely than not is not simply a white coat hypertension issue and I would like them to start treatment of some kind. However, I have several patients, for whatever reason, that are very resistant to prescription medications and who would prefer to use so-called naturopathic remedies of various types. Or just flat out refuse medications. Or say look, doc, I don't agree. I think the blood pressures that you guys are getting here are inaccurate and I'll take it myself, with my home cuff or self-monitor I would likely say well, let's meet in the middle. Why don't we do a home monitoring test and really see what they are, with a reliable method of monitoring your home blood pressures that I as your doctor feel confident in.* (Clinic 2, PCP 19)

However, PCPs and their patients did not have easy access to ABPM. Some PCPs knew the service existed but stated that ABPM was not easy to order and was not very accessible to patients. This limitation was confirmed by delivery system leaders who reported a limited number of machines and staff available to set patients up with the ABPM monitor. Other PCPs were unaware that ABPM was available to them and had never considered it an option.

*I don't think I've ever ordered a 24-hour ambulatory blood pressure reading, just because the mechanism is not that readily available. That's pretty much it. Mostly in-clinic manual blood pressure readings is what we rely on.* (Clinic 4, PCP24)

*I like the 24-hour monitor because I think it's a more realistic reading of what people are experiencing throughout the day, but I think it's a little bit of a hassle to get it set up for patients, the way that it's set up … it has to be a referral to nephrology and follow up with them, not available in primary care directly.* (Clinic 2, PCP 17)

### Patient engagement

Two important themes that were not specific to the measurement methods were patient engagement and the influence of the BP-CHECK study on provider attitudes. This section explores patient engagement and the subsequent section expands on the influence of the BP-CHECK Study. Some PCPs valued BP measurement methods that engaged patients in measuring their own BP. They stated that engagement allowed patients to better understand normal variation in BP and helped them feel empowered to manage it.

*So, the [patients] that get really into it will notice variations. So, then they will point that out. That's usually useful and helpful, especially if there are some that are on the higher side, because then that leads into showing people there is variation, it may be running higher at times when they don't realize it.* (Clinic 3, PCP2)

Respondents also acknowledged that patient engagement and patient centeredness require flexibility since patients often have different preferences, needs, and circumstances that shape how they want to and can measure their BP.

*If a patient measures blood pressure, no matter where, in the pharmacy, gas station, fire station, whatever, if a patient has a chance to measure their blood pressure, if they can write it down, I will count that.* (Clinic 6, PCP2)

*I guess patient-centered means patients have different needs …. Some patients don't want to invest in a home blood pressure monitor, they are comfortable coming into clinic and having it checked from time to time or going to a kiosk ...* (Clinic 3, PCP12)

### Influence of the BP-CHECK trial

Given that respondents interviewed for this part of the BP-CHECK study had received at least one ABPM report on a patient, they were asked if the study had affected their knowledge, beliefs or practice in any way. Most respondents did not directly attribute any changes in practice to the study, but some talked about increased knowledge, beliefs or awareness of ABPM. A few felt the study changed their beliefs and/or practices related to diagnosis, particularly in increased awareness of the availability and advantages of ABPM and the importance of variability in BP.

*…we more routinely do that second blood pressure reading and we use the automated, we use the machine. And that came out of one of the discussions about the study and the unreliability of certain techniques and certainly the manual technique is less reliable. So, we have been more consistently using the automated cuff and especially for the second blood pressure reading… I find myself more reliably asking for home blood pressure readings outside of the clinic blood pressure readings as additional information. I have ordered a couple of 24-hour ambulatory measurements since then as well.*

*Interviewer: … Is there anything that you can point to that's influenced that particular change?*

*PCP: I think the awareness of the limitation or the unreliability of the single point measurements in clinic… especially with some of our older frail adults – basing treatment on a one-point measurement, they actually increase the risk of harm if that person really doesn't show hypertension outside of clinic. So really feeling certain about the diagnosis, that the study raised awareness of certainty of the diagnosis before initiating treatment.* (Clinic 2, PCP5)

*I'm more willing to ask for and use outside readings. I've always been willing to use them, but I've also usually insisted patients still come into properly document for HEDIS [Healthcare Effectiveness Data and Information Set] purposes their results - I've got to admit I'm a little less concerned about that, and more concerned about let's make the right diagnosis and get you treated. Especially as we're trying to push the numbers down lower for good blood pressure control, I'm willing to accept more outside readings. I don't know if this ambulatory study influenced me in that decision - I suspect partly - just a willingness to be more open to use the outside readings, wherever we can get them. So that may be the only real change at this point.* (Clinic 4, PCP6)

## Discussion

Interviews with PCPs who had experience with ABPM through the BP-CHECK randomized trial revealed that overall, they preferred clinic-based BP measurement for hypertension diagnosis, especially manual BP measurement. PCPs viewed measurement in clinics as accurate because it is performed by a trained professional who can ensure optimal patient rest and body positioning. For HBPM, PCPs worried about patients’ ability to follow best practices regarding rest and body positioning, the accuracy of the monitors they have at home, and whether repeated measurements would cause patient anxiety. These results suggest that PCPs’ confidence in clinic-based BPs and concerns about HBP may be misplaced given that the main findings of the BP-CHECK study found HBPM for diagnostic testing was more accurate than clinic-based BP follow-up measurements. However, in the BP-CHECK study, patients received free validated home BP monitors and were trained to use them [16].

PCPs were also concerned that there was no acceptable way to document HBPM readings in the EHR for HEDIS purposes at the time of the interviews. (HEDIS rules have since changed to allow quality measurements to include manual and electronic entry of BP measurements taken at home.) Also, subsequent to the COVID-19 pandemic, the Center for Medicare and Medicaid Services approved use of home BP measurements for reporting quality metrics. Pursuant to this change, our health care system is testing several strategies for collecting BPs from home, including a BP telemonitoring program with BP measurements directly transferred to the EHR or patients hand-entering their home BP readings into secure email sent via the patient's shared EHR.

For kiosk-based measurements, PCPs were concerned about maintenance of the BP machines, whether testing in a public setting created stress, and patients’ ability to follow recommended resting time and positioning prior to taking measurements. PCPs also noted that kiosk results tended to be high, which aligns with the results of the main study that found kiosks but not clinic or HBPM methods tended to give higher readings than ABPM [13].

While interviewees acknowledged that ABPM is a very accurate method for measuring BP for diagnostic purposes, they correctly perceived that it was not readily available. ABPM devices are expensive and require specialized software and staff who are trained to do ABPM testing and retrieve the data. However, ABPM could be made more easily available, similar to devices that track arrhythmias that can be set up within primary clinics or mailed to patients directly. We know of only one study that is testing strategies for improving access to ABPM and home BP hypertension diagnostic testing and the results are not yet available [17]. A few PCPs acknowledged that experience with the BP-CHECK study made them more aware of ABPM as an option for hypertension diagnosis and increased their recognition of the importance and complexity of BP variation both in making a diagnosis of hypertension and helping patients understand when treatment might be necessary. Overall, PCPs stated that patient engagement and patient centeredness were important and required being flexible in the BP measurement choices offered to patients.

Our findings are consistent with and expand on prior studies that explored PCP beliefs and preferences about out-of-clinic BP measurement. One key finding from the literature comes from a systematic review of qualitative studies on patients and physicians. The studies were conducted in the U.K. (6 studies), Sweden (1), Malaysia (1), Canada (1) and the U.S. (2) and were about self-measurement of BP for management of hypertension. For hypertension management (not diagnosis), the review found that physicians: 1) recognized that that home monitoring often gave lower measurements than clinic and that created a dilemma about which BP measurements to use, 2) were concerned that HBPM would increase anxiety for patients, and 3) reported that variability in BP introduced by HBPM increased treatment uncertainty. A survey of general practitioners in the U.K. found that only a minority recognized that threshold values for diagnosis using HBPM were lower than those for clinic measurement [18, 19]. This literature points to potential barriers to PCP uptake of a variety of out-of-clinic BP measurement techniques that we also found in our research. However, our study provides a deeper dive into the views on out-of-clinic BP measurement options among PCPs in the U.S.

Our study also has a unique focus on diagnosis rather than management: Diagnosis has important implications for if and how individuals understand and begin management for their hypertension. Our findings reinforce Fletcher et al.’s themes regarding the concerns that PCPs have with self-monitored BP, including difficulties interpreting variable BP readings and reconciling them against clinic measurements as well as perhaps unfounded worries that HBPM causes patients to become overly anxious about their BP [10]. We also found that our interviewees agreed that HBPM was convenient for patients.

Our findings provide additional insight into the reliance and trust that PCPs place in clinic BP measure. PCPs in these interviews articulated a preference for in clinic BP measurement for diagnosis, which was reinforced by a parallel survey our team conducted that found that 64.7% of PCPs and 88.9% of registered nurses and MAs endorsed clinic BP as the most accurate measurement method [13]. This belief persists despite scientific evidence that other types of measurement are more accurate and more patient centered. A key finding from the main BP-CHECK study was that sensitivity of clinic BP for detecting hypertension was only 31%, while HBPM sensitivity for detecting hypertension was 81%. However, the preference for clinic BP measurement may also be due to organizational and structural factors that create real or perceived barriers to use of out-of-clinic measurement options.

Our study has many implications for practice. First, our findings highlight the need for education of both PCPs and patients about how to interpret BP values obtained by different methods using established threshold values for abnormal BP that are available for each BP measurement method. Our findings also show the importance for PCPs and patients of understanding the variability in BP and therefore the benefits of multiple readings. Indeed, PCPs appeared to lack understanding about what to do with variable readings and desire to treat BP as a more static vital sign. Our findings also show the need to find ways to demonstrate the accuracy of HBPM and increase PCP confidence in this measurement method. In addition to the need to address PCP knowledge and attitudes toward BP measurement and diagnosis, our results also show the need to address systems barriers for use of HBPM and ABPM, for example by finding secure, user-friendly mechanisms to integrate HBPM results into EHRs, ensuring that these results are acceptable for HEDIS or other quality metrics, and making ABPM more readily available to PCPs. Another policy and systems challenge that health care delivery is facing in the U.S. is that clinics and health plans are not allowed to provide patients with free home BP monitors, because they are considered to be inducements, and would violate anti-kickback laws. They can, however, loan patients home BP monitors, but getting the monitors back and cleaning them for the next patients presents additional barriers. Resources for training patients to use home BP monitors properly also might not be easily available. Overall, both increased education for PCPs regarding how best to screen for and diagnose hypertension in primary care settings and changes to clinical workflow and infrastructure are needed to improve the rate of hypertension diagnosis and treatment in the U.S.

Limitations to our study include that it was conducted in a single large, integrated healthcare system with diverse practice patterns, patients, and providers from 12 clinics. Also, the sample size,while considered robust for qualitative methods, may not have captured all possible PCP perspectives. The PCPs who participated in our study were all physicians and their results may not represent the attitudes and practice patterns of non-physicians who provide primary care within our healthcare system. As part of the study, all interviewees ere offered an educational session on BP measurement and diagnosis conducted by the study leader and had more exposure to ABPM than other PCPs not involved in the trial. We expect that PCPs who were most innovative and interested in research would volunteer to participate in the interviews; therefore, we believe this selection bias may skew our results toward PCPs who are more open to considering new methods for measuring BP and diagnosing hypertension. Finally, the results regarding patient centeredness come from the PCPs rather than patients. Our patient data will be reported in a separate manuscript.

## Conclusion

Ensuring that individuals with hypertension are appropriately identified and treated is critical to preventing cardiovascular events. Current guidelines recommend confirming elevated clinic measurements in individuals without known hypertension through out-of-clinic BP measurements to establish a diagnosis. Our findings reveal a need to improve PCP attitudes and practices regarding BP measurement for hypertension diagnosis, especially in light of recent evidence that HBPM is a more sensitive method for detecting hypertension than clinic-based measurement and now that out-of-clinic techniques are recommended for accurate diagnosis. At the same time, health systems need to invest in equipment and systems to measure and integrate out-of-clinic BP measurements into the EHR, to better facilitate the diagnosis and management of hypertension.

## Supplementary Information

**Additional file 1.** BP check physician interview guide.

## Data Availability

The authors declare that the data supporting the findings of this study are available within the article. However, additional data are available upon request from the corresponding author [CH]. The data are not publicly available since they contain information that could compromise research respondents’ confidentially that was promised in the consent process.

## Abbreviations

ABPM: Ambulatory blood pressure monitoring
BIPOC: Black, Indigenous, People of Color
BP: Blood pressure
EHR: Electronic Health Record
HBPM: Home blood pressure monitoring
MA: Medical assistant
PCP: Primary care physician
USPSTF: U.S. Preventive Services Task Force
