# Supplementary material for: Primary care physician beliefs and practices regarding blood pressure measurement: results from BP-CHECK qualitative interviews
Source: BMC Prim Care. 2023 Jan 25;24:30. doi: 10.1186/s12875-022-01950-1 (PMC9874175; doi:10.1186/s12875-022-01950-1)
Supplement: Supplementary file 1 — Additional file 1. BP check physician interview guide. [file 12875_2022_1950_MOESM1_ESM.doc]

**BP Check Physician Interview Guide**

**I. Introductions/Ice breaker**

1. Can you start by stating your role at Group Health/Kaiser Permanente Washington?

**II. Experience with BP Monitoring Since Participating in the Study**

1. Tell me about your experience or your interaction with the BP Study, so far?
   1. What activities or trainings have you been a part of?
   2. What changes have you observed in your clinic since it began participation in the study?
2. I would like you to reflect on what you do if a patient has a high BP reading in the clinic, and if there has been a change in your practice over the last [xxx months].
   1. How, if any, have you changed steps to confirm the reading?
   2. How, if any, have you changed how you decide to give someone a diagnosis of hypertension?
   3. What has it been that has made you make that change (if any?)
3. Since participating in this study, what types of BP monitoring devices do you use to make decisions about diagnosing hypertension?
   1. Have there been changes in how often have you use each type of blood pressuring monitoring device?
4. What has been the reason for the change in the devices you use to make decisions about diagnosing hypertension?
   1. Has there been a change in how you compare the different devices?
   2. Has there been a change in which you think are more or less accurate and why?
   3. Was there specific information provided to you through the study that influenced this change? Please tell me about this.
5. Since you began participation in this study, please tell me how your attitude/practice related to home BP monitoring may have changed (with an electronic BP cuff).

*Changes related to…*

- 1. Comfort with this method of BP monitoring?
  2. Types of instructions or training do you provide patients?
  3. Thoughts about the pros and cons of this method?
  4. Confidence in the readings you get using this method (accuracy)?
  5. Extent to which you use home BP monitoring to make a new diagnosis of hypertension?

1. Since you began participation in this study, please tell me how your attitudes/practice of having patients monitor their BP at a BP kiosk may have changed (the machine like the one in the lobby or found at drug stores)?

*Changes related to…*

- 1. Comfort with this method of BP monitoring?
  2. Types of instructions or training do you provide patients?
  3. Thoughts about the pros and cons of this method?
  4. Confidence in the readings you get using this method (accuracy)?
  5. Extent to which you use kiosk BP monitoring to make a new diagnosis of hypertension?

1. Can you tell me in your own words what the 24 hour ambulatory BP monitor does?
   1. How often do you order this test?
   2. If yes, what for what reasons do you order this test? If never ordered the test, why?
2. Did you receive 24 hour ambulatory BP test results for any of the patients participating in the BP-CHECK study?
   1. If so, how did you understand this information?
   2. What did you do with this information?
3. Since you began participation in this study, please tell me how your attitude/practice related to 24hr ambulatory BP monitor may have changed.

*Changes related to…*

- 1. Comfort with this method of BP monitoring?
  2. Thoughts about the pros and cons of this method?
  3. Confidence in the readings you get using this method (accuracy)?
  4. Extent to which you use kiosk BP monitoring to make a new diagnosis of hypertension?

1. Since participating in the study, what is your reflection on which method of BP monitoring you think is most patient-centered and why?
   1. Of any of the BP monitoring methods we’ve been talking about, are there some that present greater barriers to patients than others?
   2. Have you received any feedback from patients regarding their experience with BP monitoring, or about the results they have received?
2. Overall, when comparing clinic, home, kiosk, and ambulatory BP monitoring, which method of BP monitoring do you think is most accurate and why?
   1. Which do you prefer for making a new diagnosis for hypertension?
3. Overall, since your clinic began participation in this research study, what has been the biggest change for you in your practice?
   1. What about the biggest change you’ve observed in your clinic?
4. What have you found to be the most important information or aspect of this study for you in your work with patients to monitor blood pressure and diagnose hypertension?
   1. The US Preventive Services Task Force, the group that makes evidence-based screening recommendations about prevention and screening for the country, makes recommendations for measuring BP and making a new diagnosis of hypertension. What’s your understanding of current evidence-based recommendations for diagnosing hypertension?
   2. The American Heart Association and the American College of Cardiology (and endorsed by multiple other societies including the American Board of Family Medicine) recently updated hypertension diagnosis and treatment guidelines (November 2017). What’s your understanding of their recommendations for diagnosing hypertension?

Now I’d like to ask you a few questions about yourself and your training and practice. You can choose not to answer any of the questions you don’t want to answer. These questions will help us describe our work when we report to our funding agency and share our results. We will share the information only in aggregate and will not share any information such that individuals could be identified

1. What is your age?

- 18-24
- 25-34
- 35-44
- 45-54
- 55-64
- 65-74
- 75 or older

1. Are you male or female?

- Male
- Female

1. Are you of Hispanic or Latino origin or descent?

- Yes, Hispanic or Latino
- No, not Hispanic or Latino

1. What is your race? Mark one or more.

- White
- Black or African American
- Asian
- Native Hawaiian or Other Pacific Islander
- American Indian or Alaskan Native
- Other

1. How many years have you practiced medicine? _______________
2. How many years have you practiced at Kaiser Permanente Washington/Group Health? ________________
3. How many hours a week are you currently working?

- 40+ hours a week
- 39-32 hours a week
- 31-24 hours a week
- 23-20 hours a week
- Less than 20 hours a week

1. What is your specialty?

- Pediatrics
- Family medicine
- General internal medicine
- Other, please specify: _____________________________

1. Do you have anything else you would like to share with us about your experiences working with patients around BP monitoring or your clinics participation in this study?
